# Burnout, negative emotions, and wellbeing among social workers in China after community lockdowns during the COVID-19 pandemic: Mediating roles of trait mindfulness

**DOI:** 10.3389/fpubh.2022.952269

**Published:** 2022-09-14

**Authors:** Yaxue Wu, Yue Wei, Yanli Li, Jun Pang, Yang Su

**Affiliations:** ^1^Beijing Huilongguan Hospital Clinical Department III, Peking University Huilongguan Medical College, Beijing, China; ^2^Zhongke Boai (Beijing) Institute of Psychological Medicine, Beijing, China; ^3^Guangzhou Juenian Consulting Co., Ltd., Guangzhou, China; ^4^Hainan Mindfulness Education Technology Co., Ltd., Haikou, China

**Keywords:** social workers, burnout, anxiety, depression, trait mindfulness, COVID-19, wellbeing

## Abstract

**Objective:**

This study aimed to investigate burnout situation of social workers (SWs) who experienced the COVID-19 pandemic-related community lockdown 1 year before, and to assess the protective value of trait mindfulness (TM) in states of burnout.

**Method:**

We surveyed the burnout, trait mindfulness, negative emotions (NEs) and wellbeing (WB) of 182 social workers provided services to Wuhan lockdowns community by COVID-19 one year before. Burnout were measured using the Maslach Burnout Inventory–Human Services Survey; TM using the Mindful Attention Awareness Scale; NEs using the Depression Anxiety and Stress Scale-21; and WB using the General Wellbeing Schedule. We also performed correlation regression analysis and mediation test for burnout, TM, NEs, and WB.

**Results:**

Among the 182 respondents, 75 (41.2%) still suffered from severe burnout. TM was negatively correlated with burnout (r = −0.623), negatively correlated with NEs (r = −0.560), and positively correlated with WB (r = 0.617). Burnout had a significantly positive correlation with NEs (r = 0.544) and a significantly negative correlation with WB (r = −0.666). Further, WB had significantly negative correlation with NEs (r = −0.758). After controlling for age, gender, marital status, educational level, and years of employment, burnout had a significantly positive predictive effect on NEs (β = 0.509), whereas TM had a significantly negative predictive effect on NEs (β = −0.334). TM played a partial mediating role in the effect of burnout on NEs, with a mediating effect and effect ratio of 0.088 and 39.7%, respectively. Burnout had a significantly negative predictive effect on WB (β = −0.598), whereas TM had a significantly positive predictive effect on WB (β = 0.299). TM played a partial mediating role in the effect of burnout on NEs, with a mediating effect and effect ratio of −0.164 and 30.3%, respectively. WB had a significantly negative predictive effect on NEs (β = −0.711), and it played a partial mediating role in the effect of burnout on NEs, with a mediating effect and effect ratio of 0.185 and 83.3%, respectively.

**Conclusion:**

The current levels of burnout among local SWs remained high 1 year after the community lockdowns. TM played a mediating role in the relationship between burnout, NEs, and WB. Concomitantly, WB played a mediating role in the relationship between burnout and NEs. Therefore, in the context of burnout, TM is a protective factor for reducing emotional stress and risks of developing psychiatric disorders through the enhancement of WB.

## Introduction

Owing to its rapid societal developments, China has become increasingly reliant on SWs to address the welfare needs of the ever-growing grassroots societies. Correspondingly, the number of SW teams has grown rapidly over the recent years. However, the emergence of the coronavirus disease 2019 (COVID-19) resulted in a surge in the levels of occupational stress experienced by SWs. Even before the COVID-19 pandemic, SW teams in China had long been dealing with high levels of burnout and high turnover rates (1). In China, the issue of burnout among SWs has become one that requires urgent solutions. Various studies have emphasized on the crucial impact of social support on the levels of burnout among SWs (2, 3). However, the increasing lack of social support for SWs remains a major concern in Chinese societies (4). As a result, research aimed at enhancing stress tolerance among SWs is essential for ensuring sufficient levels of stress tolerance among such individuals for their protection. Therefore, this study aims to investigate the protective value of trait mindfulness (TM) in states of burnout to identify training interventions that can help in maintaining mental health among SWs in China.

## Literature review

### Burnout

Burnout is a state of physico-mental exhaustion associated with the work of providing care for others (5). The operational definition of burnout, as proposed by Maslach and Jackson (6) refers to a situation in which practitioners experience emotional exhaustion, cynicism, and low levels of self-efficacy. Burnout is a subjective feeling that arises when individuals detect significant levels of input–output occupational disparities (7). It is generally believed that various professionals, such as civil servants, teachers, and medical staff, belong to occupational groups at a high risk of burnout (8). For such individuals, burnout can be caused by increasing workloads and declining levels of work efficiency, onerous reporting systems, and burdensome paperwork, as well as various factors, such as weak organizational support structures and poor team cultures (9, 10). Current studies further show that modernized electronic health management records are becoming a new cause of burnout (11). Although burnout is yet to be classified as a mental disorder (12), an increasing number of surveys have confirmed that it is closely related to mental health issues, such as anxiety, depression, and substance abuse (13–15), thereby resulting in a corresponding deterioration in occupational wellbeing among practitioners (16).

### Current burnout situation among social workers

The responsibilities of SWs are specifically related to providing care to community residents, and the tasks of such professionals involve giving residents the assurance of access to primary healthcare, maintaining order in the community, and using their actions to maintain a “just sense of wellbeing” that transcends their roles and environments (17). The meticulous and detailed work of SWs endows them with vital social responsibilities. As a result, similar to primary healthcare workers, these professionals are highly prone to burnout (18–20). Previous studies have shown that higher burnout rates often pose a range of physico-mental health risks to practitioners in professions that involve serving people (21), and burnout is often associated with increased levels of absenteeism, higher turnover rates, and negative work attitudes (22–24).

From 2019 to date, the global transmission of COVID-19 has been prolific, and according to the World Health Organization, COVID-19 is currently classified as a “public health event of international concern” (25). In various healthcare systems across the world, a variety of approaches aimed at combating the transmission of COVID-19 continue to be implemented. Controlled studies have concluded that actively sealing off and controlling communities as well as encouraging all residents to practice home isolation play a significant role in limiting the transmission of the virus at the individual level and without the situation escalating to a social malaise, thereby containing the pandemic rapidly and effectively (25). However, the actualization of this approach requires the mobilization of substantial numbers of SWs and public healthcare personnel.

In 2020, China imposed community lockdowns owing to the COVID-19 outbreak. Throughout this period, SWs provided multiple services, including health promotion, coordinating medical treatment, and providing residents with food, medicine, and daily necessities. SWs were also required to resolve familial and psychological problems among community residents. Such problems resulted from extended periods of isolation (26). Similar to medical staff, SWs were required to bear additional workloads within short notice, and this aspect resulted in many of them experiencing psychological stress owing to an overall lack of preparation (27, 28). Currently, with the COVID-19 outbreak having been controlled at lower levels nationwide, one question remains: what are the current burnout levels among SWs who worked through a year of community lockdowns? There is currently a lack of survey data on this topic. Community management work related to COVID-19 response continued even after community lockdowns had been lifted, and the tasks were ongoing throughout that year, meaning that additional new tasks targeting community security were imposed on the initial workload among SWs. Therefore, in this study, we speculated that burnout among SWs might persist, reaching severe levels.

### Burnout, wellbeing, and negative emotions

With COVID-19 cases remaining prevalent across the world, there has been a continuous stream of reports on burnout among medical staff in many regions. These reports have indicated that burnout is often accompanied by negative emotions (NEs), such as anxiety and depression, as well as other mental health issues, including increased levels of stress, reduced wellbeing (WB), and traumatic experiences (29–31). Parallel research on burnout and various types of NEs has revealed overlapping effects between the two states, which include depression and emotional exhaustion, anxiety, and reduced professional efficacy (14). Anxiety and depression are currently included in the Diagnostic and Statistical Manual of Mental Disorders, Fifth Edition (DSM-5) diagnostic tool, and they are categorized under mental disorders (32). Additionally, there is a possibility that burnout resulting from persistently unfavorable workplace environments can result in increased incidences of mental illnesses, such as depression and anxiety, to a certain extent. Patients with mild to moderate levels of depression and anxiety disorders may not be at immediate risks of severe life hazards, such as suicide, and therefore, they can work and live independently. However, their burnout levels remain increasingly persistent, and the levels of stress they experience are highly significant compared to those of their colleagues in similar working environments (33).

Another major effect of burnout is a reduction in WB, whereby the individuals affected feel that their experience of WB in life has declined significantly (34). No link has been established between professional practitioners that serve people and those that suffer from reduced WB and increases in their occupational errors (34). However, burnout and low levels of WB have been observed as aspects resulting in reduced professional empathy and the appearance of empathy fatigue (35). In turn, professional efficiency is further hampered, thereby affecting the effective actualization of personal accomplishments (36). Some studies have established that improving WB through positive psychological interventions reduces the prevalence of depressive symptoms (37). Therefore, in this study, we speculated that WB may be a mediating variable affecting NEs when an individual is in a state of burnout.

### Social support and burnout

Rapid developments in the social work sector in China have attracted increasing social attention to the problem of burnout. Consequently, scholars have attempted to examine the factors related to burnout among SWs, and one of the factors is social support (4). Some studies have suggested that the effective work performance levels among Chinese SWs depend on the support they receive from their family, friends, team members, and especially, leaders. This corresponds to the model of interpersonal relationships in Chinese culture, which is a hierarchical structure (38). Although a stable external environment is beneficial for ensuring physico-mental health among SWs, its dependence carries specific risks. Owing to the accelerating pace of change in Chinese society, the pragmatism of pursuing benefits has gradually weakened the efforts individuals previously made to maintain workplace relationships. Existing studies have confirmed that various factors, such as poor working conditions, the lack of social support, and work–family conflicts, have become vital precursors of burnout and high turnover rates among Chinese SWs (39–41). Despite contexts involving unfavorable levels of social support, there are always some individuals who can maintain satisfactory levels of physico-mental health, and significant attention should be paid to the psychological characteristics of such individuals.

### Trait mindfulness and burnout

Mindfulness, which is a concept developed in the field of contemporary clinical psychology and healthcare, is a broad technical term related to attention and awareness. Kabat–Zinn (42) defined mindfulness as “awareness that arises through paying attention, on purpose, in the present moment, non-judgmentally.” Although scholars often describe mindfulness as a state, the pursuit of this state is not the purpose of mindfulness. Practicing the non-judgmental attitude of mindfulness and obtaining the effect of physico-mental healing is the process that guides an individual toward achieving that state (43). Such vague concepts confuse many practitioners and researchers focusing on mindfulness. Various studies on the application of mindfulness have established that the stability of the practice's long-term effects is affected by various factors among individual practitioners (44). These findings have inspired various scholars to further propose that mindfulness is akin to a psychological trait generally possessed by individuals, trait mindfulness (TM), which is jointly influenced by *a priori* endowment and *a posteriori* training (45).

Considering that the psychological characteristics of TM are something individuals may possess, the implication is that some SWs have high levels of TM, and therefore, they can maintain mild levels of burnout through self-empowerment, despite situations involving insufficient levels of external support, thereby preventing the encroachment of NEs. Previous studies related to this topic have also evaluated the impacts of psychological interventions containing elements of TM. Such interventions include enhancing participants' levels of empathy, WB, and burnout (36). TM also plays a mediating role in the relationship between social support and burnout. Various studies have concluded that TM plays such a role between perceived social support and sleep quality (46). Further, intervention projects involving TM aimed at mitigating burnout levels have established that TM, burnout, and NEs among various individuals all changed accordingly (47, 48). All the findings presented above further suggest that inter-correlations between different levels of TM, burnout, NEs, and WB exist. In this study, we further assumed that TM may play a mediating role between burnout-induced NEs and WB.

In this study, we used correlation research, regression analysis, and testing for mediating effects to verify the proposed assumptions. A survey was specifically scheduled to be conducted 1 year after the COVID-19-related community lockdowns were lifted. The purpose of this survey was to evaluate the levels of burnout among SW teams and their associated NEs and WB after they had provided a year of community services during and after the lockdowns. Meanwhile, the specific hypotheses proposed were as follows:

***H1:*** Burnout levels among SWs are positively correlated with their NEs but negatively correlated with their WB.

***H2:*** Burnout levels have a positive predictive effect on NEs but a negative predictive effect on WB, and TM has a negative predictive effect on NEs but a positive predictive effect on WB.

**Figure 1:**
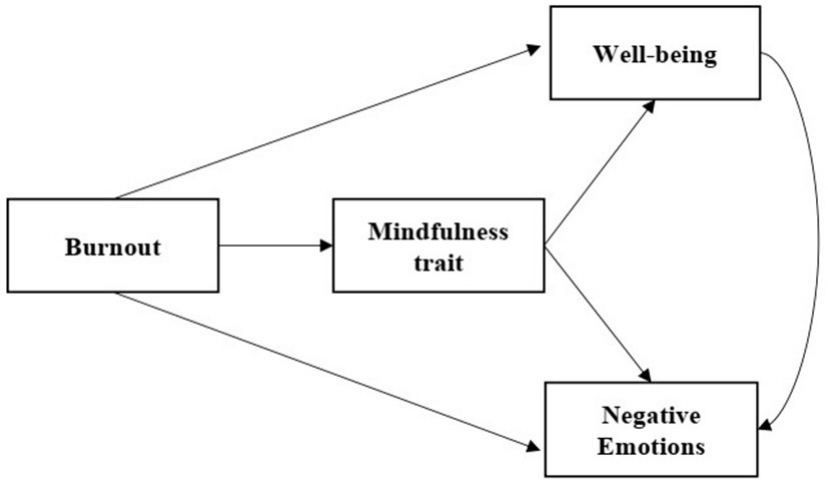
Mediating effects of TM on burnout, NEs, and WB.

***H3:*** TM plays a mediating role between burnout and NEs, and between burnout and WB, and WB plays a mediating role between burnout and NEs (Figure 1).

***H3a:*** TM plays a mediating role between burnout and NEs.

***H3b:*** TM plays a mediating role between burnout and WB.

***H3c:*** WB plays a mediating role between burnout and NEs.

## Materials and methods

### Data and samples

Data used in this study were obtained from SWs providing services to multiple communities in Wuhan, China. All the communities involved experienced lockdown enforcement during the COVID-19 outbreak in 2020. The SWs in these communities were invited to complete a questionnaire online through the support of the China Association of Social Workers in April 2021. A total of 213 questionnaires were distributed, and 182 valid questionnaires were returned, representing a response rate of 85%. There were 18 questionnaires that were not completed in time or did not meet the requirements. An informed consent procedure was implemented prior to the survey. All the respondents were informed that by responding, they would be contributing to the development of stress management services for SWs. Additionally, they were informed that their participation in the survey was voluntary and without compensation, and that they could decide to terminate their participation at their discretion.

### Method

Burnout levels were assessed using the Maslach Burnout Inventory–Human Services Survey (MBI–HSS), which is applicable to all occupations that are of service to human beings, such as those involving medical personnel and SWs. The survey's reliability and validity have been proven to be satisfactory after being applied in different countries and industries (49). The original MBI–HSS comprises a total of 22 items. The Chinese version, which has been simplified to 17 items for clinical use, was applied in this study. It contains three dimensions: (i) emotional exhaustion (7 items, e.g., “At work, I feel that my emotions are that of exhaustion”); (ii) depersonalization (3 items, e.g., “I have become increasingly indifferent to other people ever since starting this job”); and (iii) personal accomplishment (7 items, e.g., “I can solve problems at work effectively”).

A 7-point Likert scale, ranging from 0 (almost never) to 6 (almost always), was used for scoring. The dimension of personal accomplishment was reverse-scored. The higher the total score was, the more severe the individual's burnout level was. The Cronbach's alpha coefficient of the MBI–HSS (Chinese version) was 0.88 (50). The possible scores ranged from 0 to 102. For the score of 50 in demarcated burnout severity, a score <50 indicated mild burnout, whereas a score of ≥50 indicated severe burnout for which psychological intervention was necessary.

TM was assessed using the Mindful Attention Awareness Scale (MAAS), which was developed by Brown and Ryan (51). The 15 items cover only one dimension. The performance of TM over the immediate preceding week was measured using a 6-point Likert scale, ranging from 1 (almost always) to 6 (almost never). All the questions were reverse-scored: The higher the scores, the higher the individual's TM level. The Chinese version of the scale has been used widely, and it has high reliability and validity, with a Cronbach's alpha coefficient of 0.87 (52).

NEs were assessed using the Depression Anxiety and Stress Scale-21 (DASS-21). The scale's total number of items was reduced from the original 42 to 21 items, thereby covering three dimensions, each with seven items: (i) depression (e.g., “I cannot seem to experience any positive feelings at all.”); (ii) anxiety (e.g., “I do not have any valid reason to be afraid.”); and (iii) stress (e.g., “I find it difficult to relax.”). A 4-point Likert scale was used for scoring, which ranged from 0 (never descriptive of me) to 3 (always descriptive of me). The higher the score, the more severe the individual's NEs.

The psychometric properties of the DASS-21 have been proven to be satisfactory through a comparative study of this scale using the Beck Depression Inventory (BDI) and the Beck Anxiety Inventory (BAI). The correlation between the DASS-21 and the BAI was 0.81, and that between the DASS-21 and the BDI was 0.74 (53). The DASS-21 has also been validated throughout its applications in China and Hong Kong (54). The Cronbach's alpha coefficients of the three dimensions in the Chinese version were 0.83, 0.80, and 0.82, respectively. The Cronbach's alpha coefficient of the total score was 0.92 (55).

WB was assessed using the General Wellbeing Schedule (GWB) (56), which is a short, reliable, and valid measure of WB. The GWB comprises 33 items covering six dimensions: (i) satisfaction and confidence in life, (ii) worries about health, (iii) energy level, (iv) depressive or happy moods, (v) control over emotions and behavior, and (vi) relaxation and tension. The scores for each item range from 0–3 to 0–10, with a higher overall score indicating higher levels of WB. The Chinese version of the GWB has satisfactory psychometric properties, with the Cronbach's alpha coefficients of the scale being 0.91 for males and 0.95 for females (57).

### Analysis strategy

All analyses were performed using the Statistical Package for the Social Sciences (SPSS) 26.0 software. First, descriptive analyses and correlation tests were performed on the total score of each scale. Next, the various variables were subjected to linear regression analysis. The MAAS scores were considered the independent variables. The total scores of the MBI-HSS, DASS-21, and GWB represented the dependent variables, and the control variables included gender, age, marital status, educational level, and years of employment. The SPSS-PROCESS plug-in was used to verify the mediating effect of the MAAS on the regression between the MBI-HSS and the DASS-21 and that between the MBI-HSS and the GWB.

## Results

Data used in this study might have been subject to common method bias because these data were obtained from self-reports filled by the respondents. Harman's one-factor test revealed that three factors had eigenvalues >1. The first factor explained 31.98% of the variance, which was less than the critical value of 40%. Therefore, it was deemed that the data had significant levels of common method bias.

The respondents comprised 18 males (9.9%) and 164 females (90.1%). They were aged between 19 and 60 years. The median age was 42 years, and the mean ± standard deviation was 41.09 ± 9.41 years. In terms of marital status, 126 (69.2%) were married, 34 (18.7%) were unmarried, and 22 (12.1%) were divorced/separated. Regarding educational qualifications, 11 (6.0%) had completed high school/technical secondary school, 44 (24.2%) had attended a professional training college, 101 (55.57%) held bachelor's degrees, and 26 (14.3%) had a master's degree or above. More than half of the respondents had worked for more than a decade: 62 (34.1%) and 120 (65.9%) had ≤ 10 and >10 years of employment, respectively.

The average scores of the sample were as follows: 36.90 (SD = 12.54) for burnout, 51.52 (SD = 10.62) for TM, 34.14 (SD = 7.29) for NEs, and 71.74 (SD = 15.12) for overall WB. Taking the total MBI–HSS score of 50 as the demarcation point, it was established that 75 (41.2%) of the respondents suffered from severe burnout. The various descriptive statistics of the demographic characteristics and the key variables of the two groups of the sample with mild and severe burnout, as well as a comparison between the related factors, are listed in Table 1. The data indicated that SWs who were older and had been employed longer generally had milder burnout levels. In terms of marital status, the proportion of married respondents in the mild burnout group was significantly higher. The TM and WB scores were higher among the mild burnout group, and their NEs scores were lower.

**Table 1:** General characteristics of respondents in the mild vs. severe burnout groups and a comparison of related factors.

	**Mild burnout (MBI–HSS <50)**	**Severe burnout (MBI–HSS ≥50)**	***t*/χ^2^**	***p-*value**
	**(*n* = 107)**	**(*n* = 75)**		
**Gender [N (%)]**
Male	9 (8.4)	9 (12.0)	0.637	0.425
Female	98 (91.6)	66 (88.0)		
**Age (M** **±SD)**	43.11 ± 8.76	38.21 ± 9.60	3.568	0.000*
**Marital status [*****N*** **(%)]**			6.398	0.041*
Married	77 (72.0)	49 (65.3)		
Not married	14 (13.1)	20 (26.7)		
Divorced/separated	16 (15.0)	6 (8.0)		
**Educational level [*****N*** **(%)]**			3.244	0.356
High school/ technical secondary school	5 (4.7)	6 (8.0)		
Professional training college	30 (28.0)	14 (18.7)		
Bachelor's degree	59 (55.1)	42 (56.0)		
Master's degree or above	13 (12.1)	13 (17.3)		
**Years of employment [*****N*** **(%)]**
≤ 10	30 (28.0)	32 (42.7)	4.201	0.040*
>10	77 (72.0)	43 (57.3)		
**TM (M** **±SD)**	56.19 ± 8.78	44.87 ± 9.42	8.304	0.000*
**NEs (M** **±SD)**	31.47 ± 5.90	37.96 ± 7.42	−6.568	0.000*
**WB (M** **±SD)**	78.60 ± 12.09	61.96 ± 13.58	8.681	0.000*

N = 182, ^*^p < 0.05.

The correlations among the key variables of TM, burnout, NEs, and WB are listed in Table 2. A Pearson's correlation test demonstrated that TM was negatively correlated with burnout (*r* = −0.623, *p* < 0.01) and NEs (*r* = −0.560, *p* < 0.01), but it was positively correlated with WB (*r* = 0.617, *p* < 0.01). Although burnout was positively correlated with NEs (*r* = 0.544, *p* < 0.01), it was negatively correlated with WB (*r* =-0.666, *p* < 0.01). WB was negatively correlated with NEs (*r* = −0.758, *p* < 0.01). Therefore, **H1** was verified.

**Table 2:** Correlation matrix among the key variables of TM, burnout, NEs, and WB.

	**Variable**	**Mean (SD)**	**1**	**2**	**3**	**4**
1	TM	51.52 (10.62)	1			
2	burnout	49.19 (16.72)	−0.623*	1		
3	NEs	34.14 (7.29)	−0.560*	0.544*	1	
4	WB	71.74 (15.12)	0.617*	−0.666*	−0.758*	1

N = 182, ^*^p < 0.01.

Next, a regression analysis was conducted on burnout, TM, NEs, and WB while controlling for age, gender, marital status, educational level, and years of employment. Concomitantly, we examined whether TM played a mediating role between burnout, NEs, and WB. The results of the regression analysis and the mediating effects of TM on burnout and NEs are listed in Table 3.

**Table 3:** Mediating effects of TM on burnout and NEs.

	**NEs**			**TM**			**Burnout**		
	**β**	**SE**	***p-*value**	**β**	**SE**	***p-*value**	**β**	**SE**	***p-*value**
Age	−0.204	0.062	0.012*	0.190	0.084	0.011*	−0.141	0.060	0.073
Gender	−0.012	1.531	0.847	−0.061	2.072	0.295	−0.033	1.465	0.588
Marital status	−0.121	0.677	0.064	0.051	0.916	0.404	−0.104	0.647	0.095
Educational level	−0.119	0.588	0.054	0.082	0.795	0.151	−0.092	0.564	0.122
Years of employment	0.092	0.503	0.238	−0.213	0.681	0.003**	0.020	0.492	0.788
Burnout	0.509	0.028	0.000**	−0.606	0.038	0.000**	0.307	0.034	0.000**
TM							−0.334	0.053	0.000**
*R* ^2^	0.343			0.433			0.406		
Adjusted *R*^2^	0.320			0.413			0.382		
*F* value	*F*_(6, 175)_ = 15.212**			*F*_(6, 175)_ = 22.266**			*F*_(7, 174)_ = 16.985**		

N = 182, ^*^p < 0.05, ^**^p < 0.001.

Burnout had a positive predictive effect on NEs (β = 0.509, *p* < 0.001), even after the addition of TM as a mediating variable (β = 0.307, *p* < 0.001), whereas TM had a negative predictive effect on NEs (β = −0.334, *p* < 0.001). TM played a partial mediating role in burnout's impact on NEs, with a mediating effect of 0.088, 95% CI [0.102-0.305], and an effect ratio of 39.7%.

The mediating effects of TM on burnout and WB are listed in Table 4. Burnout had a significantly negative predictive effect on WB (β = −0.598, *p* < 0.001), and TM had a significantly positive predictive effect on WB (β = 0.299, *p* < 0.001). TM played a partial mediating role in burnout's impact on NEs, with a mediating effect of−0.164, 95% CI [-0.285-−0.090], and an effect ratio of 30.3%.

**Table 4:** Mediating effects of TM on burnout and WB.

	**WB**			**TM**			**WB**		
	**β**	**SE**	***p-*value**	**β**	**SE**	***p-*value**	**β**	**SE**	***p-*value**
Age	0.320	0.112	0.000**	0.190	0.084	0.011*	0.263	0.108	0.000**
Gender	−0.011	2.753	0.845	−0.061	2.072	0.295	0.008	2.623	0.882
Marital status	0.077	1.217	0.176	0.051	0.916	0.404	0.061	1.158	0.254
Educational level	0.040	1.057	0.456	0.082	0.795	0.151	0.015	1.010	0.766
Years of employment	−0.121	0.904	0.074	−0.213	0.681	0.003	−0.057	0.880	0.387
Burnout	−0.598	0.050	0.000**	−0.606	0.038	0.000**	−0.417	0.060	0.000**
TM							0.299	0.095	0.000**
*R* ^2^	0.506			0.433			0.557		
Adjusted *R*^2^	0.489			0.413			0.539		
*F* value	*F*_(6, 175)_ = 29.906**			*F*_(6, 175)_ = 22.266**			*F*_(7, 174)_ = 31.259**		

N = 182, ^*^p < 0.05, ^**^p < 0.001.

**Figure 2:**
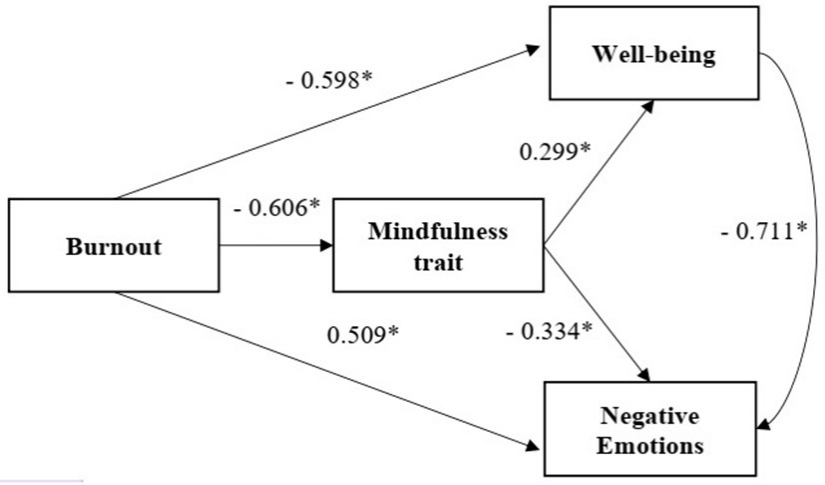
Mediating effect of MAAS on the impact of MBI–HSS on DASS-21 and GWB. Standardized β, *N* = 182, * *p* < 0.05.

The mediating effects of WB on burnout and NEs are listed in Table 5. WB had a significantly negative predictive effect on NEs (β = −0.711, *p* < 0.001). Further, when WB was used as the mediating variable, the positive predictive effect of burnout on NEs was weakened (β = 0.084, *p* > 0.01). WB also played a partial mediating role in burnout's effect on NEs, with a mediating effect of 0.185, 95% CI [0.313–0.545], and an effect ratio of 83.3%. This mediating relationship is presented intuitively in Figure 2. Based on the results presented above, **H2** and **H3** were verified.

**Table 5:** Mediating effects of WB on burnout and NEs.

	**NEs**			**WB**			**NEs**		
	**β**	**SE**	***p-*value**	**β**	**SE**	***p-*value**	**β**	**SE**	***p-*value**
Age	−0.204	0.062	0.012*	0.320	0.112	0.000***	0.023	0.052	0.730
Gender	−0.012	1.531	0.847	−0.011	2.753	0.845	−0.020	1.210	0.691
Marital status	−0.121	0.677	0.064	0.077	1.217	0.176	−0.067	0.538	0.197
Educational level	−0.119	0.588	0.054	0.040	1.057	0.456	−0.091	0.465	0.064
Years of employment	0.092	0.503	0.238	−0.121	0.904	0.074	0.006	0.401	0.924
Burnout	0.509	0.028	0.000***	−0.598	0.050	0.000***	0.084	0.028	0.201
WB							−0.711	0.033	0.000***
*R* ^2^	0.343			0.506			0.592		
Adjusted *R*^2^	0.320			0.489			0.576		
*F* value	*F*_(6, 175)_ = 15.212***			*F*_(6, 175)_ = 29.906***			*F*_(7, 174)_ = 36.117***		

N = 182, ^*^p < 0.05, ^**^p < 0.001.

## Discussion

The issue of burnout among medical staff received widespread attention during the pandemic. However, relatively few reports have examined burnout among SWs, who are also at a significantly high risk of experiencing psychological stress in the workplace. Existing surveys have concluded that burnout levels among SWs are increasing globally (58, 59). The findings of this study indicate that SWs still had high levels of burnout 1 year later after serving in communities that were under lockdown measures to control the COVID-19 pandemic in 2020. Of the total survey population, 41.2% of the respondents had a severe level of burnout. Contrarily, according to a survey conducted by Peng Shiyue et al. (60) in March 2020, which involved 249 medical staff in Wuhan who were similarly involved in COVID-19 prevention and control work, only 24.9% of the surveyed population had burnout. This proportion was much lower than that in the data gathered in this study and certainly lower than that in the data collected in surveys involving medical practitioner counterparts in other countries during the same period (30, 61, 62).

The relatively low burnout levels among the medical staff in China might be related to the rapid control of COVID-19 in Wuhan owing to the lockdowns enforced. However, SWs did not benefit from any reduction in burnout levels as a result of those measures. Although medical staff had significant levels of direct contact with infected patients, they received sufficient prior training and proper protective equipment. Therefore, their risk of infection was relatively low, and their confidence to complete their tasks was high. Medical staff across China has perennially managed heavy workloads and are often in a state of pessimism regarding their career prospects (63). They also suffer from significant levels of burnout owing to the persistent hazard of doctor–patient disputes and the long-standing negative evaluations that they face online and on social media (64, 65). Although the sudden outbreak of the epidemic has put enormous pressure on China's medical industry, the medical staff have received active publicity and social support, which has enhanced their sense of personal achievement and professional enthusiasm.

Although the sudden outbreak of the COVID-19 epidemic exerted enormous pressure on China's medical industry, the medical staff have received active publicity and social support, which has enhanced their sense of personal achievement and professional enthusiasm. This phenomenon of a high sense of personal achievement despite the pressure is also found in the Serbian doctor group (66). Since personal achievement is inversely proportional to emotional exhaustion, it is speculated that the level of burnout among Chinese physicians may have been offset to some extent by higher personal achievement, and may actually have been reduced.

Surveys conducted in Europe have indicated that professionals with non-medical backgrounds (e.g., psychologists, Pharmacists, SWs, et al.) might have faced additional stress levels compared to medical staff when undertaking work associated with COVID-19 prevention and control (66, 67). The tasks performed by medical staff in such situations were relatively specific, whereas those performed by SWs were highly complicated, menial, and nuanced (68). SWs also had to directly manage the actual difficulties, complaints, and mental health of community residents during the lockdown periods (69). These factors might have caused SWs to accumulate additional psychological pressure and trauma during the pandemic. In the 1 year since the pandemic was brought under control in China, long-term pandemic prevention and control work is still being conducted in the communities, which has significantly increased workload among SWs compared with their workload before the COVID-19 outbreak. Furthermore, most of them did not receive any prior training regarding stress reduction or recovery from psychological trauma. Therefore, we deduced that the aforementioned psychological problems they faced had not been alleviated effectively.

In addition to external work pressure, another source of burnout among SWs might be their personal characteristics, such as compartmentalization of their emotions and the inability to recognize the sources of such emotions and feelings of pressure. Using a cross-sectional survey as its basis, this study's focus was the impact of SWs' TM (a psychological quality) on their burnout, NEs, and WB. The findings indicate that TM is negatively correlated with both burnout and NEs in stressful contexts, whereas it is positively correlated with WB. A regression analysis further confirmed that burnout promoted the generation of NEs and weakened WB, whereas TM still promoted WB and ameliorated the generation of NEs in the context of burnout. A similar phenomenon has been identified in various studies, such as those conducted by Sala et al. (70), Dillard and Meier (71), Trombka et al. (72), and Nadler et al. (73). When testing the mediating effects in this study, we established that burnout levels significantly reduced individuals' WB and correspondingly, increased their risk of NEs. TM played a partial mediating effect. When burnout levels were at severe levels, individuals with higher levels of TM had relatively better WB and fewer NEs. It is worth mentioning that WB was also found to have a partial mediating effect on burnout's impact on NEs.

Combining our findings with those of previous studies, the following psychological process of stress was inferred: When individuals experience an excessive stress load, their first response is physical and mental burnout as well as an instinctive resistance to the stressful occupational environment, which is followed by a decline in their levels of professional enthusiasm. If there is insufficient social support at this time and the individuals lack adequate self-support, their WB will decline, thereby resulting in increased levels of NEs, such as anxiety and depression. Individuals with low levels of TM tend to have lower self-compassion, thereby intensifying the physical and mental reactions of NEs (74). Eventually, their ability to function in social contexts will be affected, and their condition will satisfy the diagnostic criteria for mental illness. Some of the negative behaviors, such as complaints and impulsiveness, arising from the NEs will further affect the overall workplace experience and result in increased levels of burnout (75).

The core idea of TM involves having the self-awareness to truly experience the present moment consciously and non-judgmentally. Individuals with higher levels of TM are more focused on the awareness and self-acceptance of their physical and mental feelings, thereby reducing the risks of mental rumination and emotional dysregulation and enhancing their perceptions of social support (4), which results in an effective increase of WB in their minds (76). The occurrence of burnout can be prevented through the influence of various mediating effects. In the early burnout stages, these mediating effects can also protect individuals' WB and reduce their risk of developing anxiety and depression.

The inferences presented above are supported by the corresponding research data. For example, at the physiological level, individuals with high levels of TM have lower levels of cortisol and higher levels of parasympathetic nerve excitation. They also feel less burnout in stressful environments and have an increased tolerance to physical and mental discomfort (77, 78). At the psychological level, the non-judgmental attitude associated with TM makes individuals more inclined toward internal attribution. Previous studies have confirmed that such internal attribution improves self-efficacy among individuals (79), and it has been proven to be a protective factor against burnout (80). With a non-judgmental attitude, individuals with higher levels of TM are more likely to react by engaging in positive cognitive reconstruction and enhancing their subjective initiative when they deal with real stress in workplace environments (79). Therefore, such individuals can comprehend all their work-related problems from a new and highly macroscopic perspective, thereby attaining significant levels of WB (81). These findings indicate that TM is closely related to sufficient physical and mental health (70).

In this study, we focused on examining the practical value of TM in alleviating the negative physico-mental experiences resulting from burnout, and we constructed a mediating relationship model among the burnout, TM, NEs, and WB levels of SWs in China. The findings provide further insight into the current status of SWs' occupational stress when engaging in COVID-19 prevention and control, and they contribute to the literature supporting the examination of the value of TM among SWs' professional lives. Nevertheless, the findings have certain limitations. First, the results were based on data obtained from a cross-sectional survey. When communities in Wuhan were urgently locked down as a result of the COVID-19 pandemic, SWs had neither the time nor the conditions to participate in such surveys. Valuable data could have been obtained if such a survey was conducted a year earlier, and this is a regrettable situation. Second, the data were mainly collected through the subjective reporting of SWs, and the sample size was relatively small. The coverage was incomplete, and there might have been sampling bias. Despite the limitations mentioned above, this study highlighted the importance of individual intrinsic psychological qualities on mental health among SWs. The results serve as a reference for governments and the global society at large to work toward improving the occupational welfare of SWs. In our future studies, relevant training frameworks will be developed to enhance TM among individuals. We shall also further verify the actual effects of TM enhancement on reducing burnout levels through interventional research.

## Conclusion

The findings of this study confirmed that the burnout levels among SWs in China remained relatively high 1 year after the enforcement of COVID-19 related community lockdowns. TM played a mediating role in the relationship between burnout levels, NEs, and WB, whereas WB played a mediating role between burnout levels and NEs. In states of burnout, TM, through the improvement of WB, acted as a protective factor that reduced emotional stress and the risk of developing psychiatric disorders among SWs in China.

## Data availability statement

The raw data supporting the conclusions of this article will be made available by the authors, without undue reservation.

## Author contributions

YWu was responsible for the design of the study, the processing and analyses of the data, and the writing of the paper. YWe and YL were responsible for technical guidance and the quality control of the study. JP and YS were in charge of data collection. All authors have read and approved the final manuscript.

## Funding

The funding required for this study was fully covered by the following scientific research funds: (1) Research project under the Capital Clinical Characteristics Application Program: Quantitative evaluation and standardized research on the efficacy of mindfulness-based cognitive therapy in the treatment of depressive disorders (Z171100001017103). (2) Development program of the Beijing Municipal Administration of Hospitals: Randomized controlled study to evaluate the efficacy and safety of mindfulness-based cognitive therapy at relieving anxiety and depressive symptoms and prevention of relapse in patients with bipolar disorder (PX2020076).

## Conflict of interest

Author JP was employed by Guangzhou Juenian Consulting Co., Ltd. Author YS was employed by Hainan Mindfulness Education Technology Co., Ltd. The remaining authors declare that the research was conducted in the absence of any commercial or financial relationships that could be construed as a potential conflict of interest.

## Publisher's note

All claims expressed in this article are solely those of the authors and do not necessarily represent those of their affiliated organizations, or those of the publisher, the editors and the reviewers. Any product that may be evaluated in this article, or claim that may be made by its manufacturer, is not guaranteed or endorsed by the publisher.

## Abbreviations

SW: social worker
TM: Trait mindfulness
NEs: Negative emotions
WB: Wellbeing
COVID-19: Coronavirus disease-19.
